# Chronic Mercury Poisoning From Daily Cosmetics: Case Report and Brief Literature Review

**DOI:** 10.7759/cureus.19916

**Published:** 2021-11-26

**Authors:** Zhongying Wang, Xiang Fang

**Affiliations:** 1 Medicine, Heze Medical College, Heze, CHN; 2 Neurology, University of Texas Medical Branch, Galveston, USA

**Keywords:** behavioral changes, pain, muscle atrophy, cosmetics, mercury poisoning

## Abstract

Chronic mercury poisoning from cosmetics is often misdiagnosed and mistreated due to atypical clinical presentations and high industrial standards and quality control of cosmetic products. Here we present a case of a 45-year-old female with a four-month history of progressive weakness, atrophy, insomnia, mood swings, chorea-like movement, extremity pain and hyperalgesia. The routine workup for neuropathy and myopathy such as CK, EMG were unremarkable. However heavy metal screen revealed significantly elevated mercury levels. Afterwards, we found it was caused by daily-use whitening cosmetics through the hair segmented toxicant analysis to trace the dynamics changing of mercury concentration in the body. After the removal of the patient from the source of exposure, and chelation therapy, her symptoms had gradually improved. When patients have unexplained behavioral changes, cognitive decline, sleep disturbance, fatigue, pain, and other unspecific manifestations, the possibility of mercury poisoning should be considered, blood mercury and urine mercury should be detected, and the source of exposure should be investigated and timely treatment should be given.

## Introduction

Mercury occurs naturally in the earth’s crust, but human activities, such as mining and fossil fuel combustion, have led to widespread mercury pollution. Mercury emitted into the air eventually settles into water or onto land where it can be washed into surrounding water. Once deposited, certain microorganisms can change it into methylmercury, a highly toxic form that builds up in fish, shellfish and animals that consume fish. Acute and chronic mercury poisoning caused by the occupational environment are not uncommon as well, but chronic occult mercury poisoning is often unrecognized. Because different organs and tissues have different responsiveness to mercury, the clinical manifestations of chronic mercury poisoning are extremely variable, and the signs are also not specific, which can easily lead to misdiagnosis and delay of the treatment. The patient we reported here suffered from the sensory, motor, and autonomic dysfunction caused by using daily cosmetics.

## Case presentation

A 45-year-old female was admitted to our hospital due to "progressive fatigue and limb pain for more than 4 months". More than four months ago, she had limb weakness, feet numbness, and pain without obvious triggers. The symptoms were worse after the activity, and the symptoms gradually increased to the point that she could not even tolerate a short distance of walking. She felt difficult to stand up after squatting, with stiff knees and pain in the lower abdomen and hip. The patient had paroxysmal general discomfort, irritability, anxiety, tossing and turning, involuntary dancing of both upper limbs, compulsive flexion and extension of both lower limbs, paroxysmal crying, and frantically rubbing hips. The above symptoms could last about 15-60 minutes and the pain could be relieved slightly after massaging the limbs. The patient also reported hyperhidrosis or profuse sweating, and occasionally headache and nausea during the course of the disease, which occurred when she woke up in the morning. She went to several local hospitals and was diagnosed with a "functional disorder" since no organic causes were identified. The lab and extensive work-up didn’t show any abnormalities such as thyroid function test, inflammation indexes, rheumatic immune indexes, electromyography (EMG/NCS) and CT scans (the results showed lumbar spine showed L3/4, L4/5, and L5/S1 disc bulging), and her symptoms did not show any significant improvement after prescribed NSAIDs and alternative treatment (i.e., massage, acupuncture and cupping). The patient came to our department for further evaluation and treatment. Since the onset of the symptoms, she complained of poor sleep, fatigue, and decreased appetite. During the past two months, she slept about 2-3 hours intermittently every day. Her bowel movements were normal, and she had lost 7 kilograms in the past four months.

Physical examinations of vital signs were within normal limit, her BMI was 17.69. She was alert with poor concentration, anxious and painful appearance, flushing of cheeks and eyes, difficulty walking, staggering gait, and tongue trembling. She felt hyperalgesic all over the body, especially in the lower limbs and feet, and felt unbearable pain when a cotton swab was lightly scratched on the local skin. She has significant muscle atrophy of both lower extremities. The proximal muscle strength of both lower extremities was Grade 3, the tendon reflex was basically normal, and the right side Chadok sign was (+). The others showed no obvious abnormalities.

**Figure 1:**
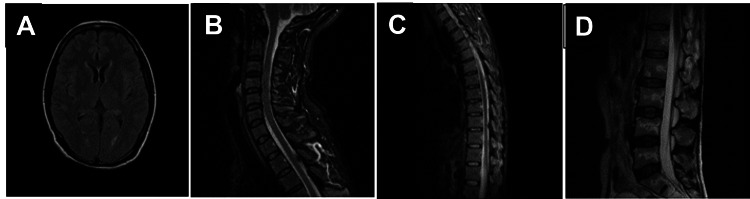
Neuro-axis imaging: (A) brain; (B) cervical spine; (C) thoracic spine; (D) lumbar spine.

Repeated CBC, CMP, CK, TSH, free T4, ESR, RF, RPR, vitamin B1, B6, B12, and tumor markers were normal. Brain and spinal MRI (Figure 1) did not show abnormality except bulging disk without spinal canal stenosis or foraminal narrowing. Ultrasound of abdomen and pelvic did not show any mass lesion and EMG/NCS including peroneal, tibial, median and ulnar motor nerve conduction, sural, median and ulnar sensory nerve conduction, medial tibial F wave, and tibial nerve H-Reflex, tibialis, Medial gastrocnemius EMG was within normal limits (data not shown).

During the hospitalization, she was given symptomatic treatments such as anxiolytic, antidepressant and nutritional supplement. However, her symptoms did not improve, and she lost 6 kg in one month after admission. Taking into account that she worked in galvanized iron sheet factory, we conducted a toxicant test on the patient’s urine to exclude the poisoning caused by physical or chemical intoxication. The results showed that urine mercury level was significantly increased, reaching 16.0 µg/g (Shandong Provincial Occupational Disease Hospital, the biological exposure limit is 35 µg/g, occupational mercury exposure limit should be <5 µg/g). After that, we sent urine again and at the same time sent blood and hair for a heavy metal test to Beijing Gaoxin Borui Quality Inspection Technical Service Co., Ltd. The results showed that the mercury content of both blood and hair in the tested samples exceeded the standard. The results are shown in Table 1.

**Table 1:** Test results of mercury content in blood, hair and urine.

Samples	Blood (ng/ml)	Reference range (ng/ml)	Hair (ng/mg)	Reference range (ng/mg)	Urine (ng/ml)	Reference range (ng/ml)
Mercury (Hg)	7.8	＜2.5	9.3	＜1.56	10.3	＜15
Lead (Pb)	7.6	＜29.0	1.5	＜10.39	6.4	＜70
Arsenic (As)	8.7	＜31.6	0.2	＜1.03	11.3	＜100
Thallium (Ti)	0.07	＜0.1	0.02	＜0.1	0.07	＜5

**Figure 2:**
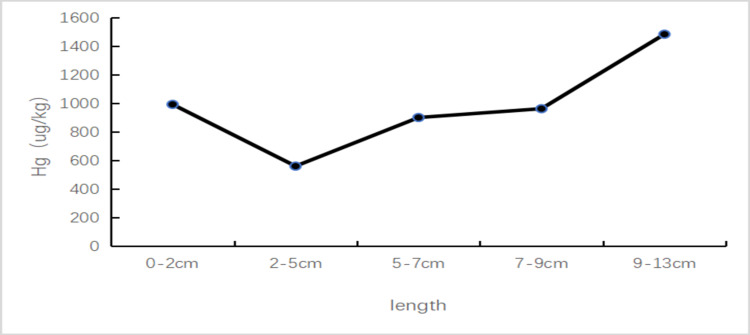
Schematic diagram of mercury concentration changing with hair length (Fuda Analytical Testing Group, Shanghai, China, The normal value in this laboratory is <300 µg/kg).

Because the patient was engaged in custody and financial work, she wouldn’t contact the galvanized iron sheet directly, and her husband and other workers who contact the galvanized iron sheet directly had no similar symptoms, so the disease caused by the occupational environment was unlikely. In order to further identify the source of the poison and the mercury concentration changes in the hair in the past six months, a strand of proximal hair of about 13 cm, was taken. According to the test results and the hair growth rate of 1-1.5 cm per month, it could be estimated that the mercury content in the measured hair would be the highest from July to September 2020, and there was a trough from November to December 2020, and then it rose again. The changing process is shown in Figure 2. Following this pattern, we asked the patient about her daily contacts and environmental history. After careful recollection, she recounted that she purchased a kind of cosmetic online about half a year ago (May 2020). Her symptoms started after using it for more than two months. Since the symptoms were mild and this did not bring to her attention. She began to see a doctor in September 2020 due to severe symptoms. Her condition was severely affecting her daily life, so she rarely used those cosmetics from November to December of that year, then the symptoms were slightly relieved. However, she resumed using them again and the symptoms worsened. This cosmetic was mailed to a Beijing poison testing center, and the result showed that the mercury content was 9856.2 µg/g (allowable limit value <1µg/g).

After the diagnosis of mercury poisoning was confirmed, she was given dimercaptosuccinic acid capsules orally to repel mercury. Unfortunately, she felt nausea, vomiting, and got severe gastrointestinal reactions, so we prescribed her Sodium Dimercaptopropane Sulfonate 0.125 g/day (intramuscular injection, using three days and stopping four days as a cycle) and instructed her to drink more water to help mercury excretion from the body. One month after the mercury excretion treatment, her blood mercury concentration dropped to 3.1 ng/mL (the reference range was ＜2.5 ng/mL), and her symptoms gradually improved, she even gained 2 kg from the previous treatment.

More than a month after discharge, she was readmitted to our hospital for the second course of treatment with the same regimen as before. After four cycles, all of her symptoms resolved, and she was discharged on June 13, 2021. On her follow-up visit on October 21, 2021, she complained of mild insomnia and short-term memory impairment, but without sensory and motor symptoms.

## Discussion

We present a unique case of mercury intoxication caused by cosmetics skin cream. Due to insufficient inquiry and differential diagnosis, this patient was misdiagnosed as having vertebral body disease and mental disease by different hospitals. In addition, the lack of understanding of mercury poisoning and peripheral neuropathy by clinicians has led to a high rate of misdiagnosis of the disease. The possibility of mercury poisoning should be considered when patients have unexplained impatience, memory loss, sleep disturbance, fatigue, pain, and other unspecific manifestations, and blood mercury and urine mercury should be checked, and timely treatment should be given.

Mercury is a highly toxic heavy metal with three forms: metallic mercury (elemental mercury), inorganic mercury (mercury salt), and organic mercury compounds. Different forms of mercury have different toxicological characteristics. Mercury can be absorbed into the human body by inhalation, ingestion, dermal absorption, or injection. Human exposure occurs mainly through inhalation of elemental mercury vapours during industrial processes, and through the consumption of contaminated fish and shellfish, which is called Minamata disease [1,2]. Skin is the most important application for cosmetics. Inorganic mercury can be absorbed via the sweat glands, sebaceous glands, and hair follicles, and after absorption, it is distributed to all tissues. Repeated topical applications can result in systemic toxicity, including kidney damage and nervous system [2]. Long-term exposure to mercury-containing preparations can cause nephrotic syndrome, and the most common renal pathological patterns reported in the literature are MN and minimal change disease [3]. We have performed a literature meta-analysis using keywords such as mercury poisoning/toxicity, cosmetics, lotion, skin etc. Fifty-six literature are identified, 521 people including 516 women and five men were reported. The age of the patients ranged from 17-month-old to 57 years old and the time of using or contacting cosmetics ranged from 15 days to 15 years. In all clinical manifestations, the proportion of nonspecific symptoms were as follows: insomnia 58.9% (307/521), dizziness 54.9% (286/521), headache 39.5% (206/521), fatigue 36.7% (191/521), hypomnesia 25.7% (134/521). Mental system abnormalities were mainly emotional changes, and the predominant manifestations of kidney injuries were increased urinary protein. Digestive system symptoms were mostly anorexia and nausea, and nervous system damages were mostly pain, numbness, hypoesthesia and tremor. Skin changes were mainly manifested as skin rash and skin redness, cardiovascular abnormalities were mainly hypertension and rapid or slow heart rate, and endocrine system abnormalities were mainly hypothyroidism. Among these patients, 33 patients had nervous system symptoms dominated by pain, but only 15 underwent neuroelectrophysiological examination, of which four were abnormal. Therefore, for patients with unexplained pain in young women, especially the neuroelectrophysiological examination results are normal, the possibility of mercury poisoning should be considered.

**Figure 3:**
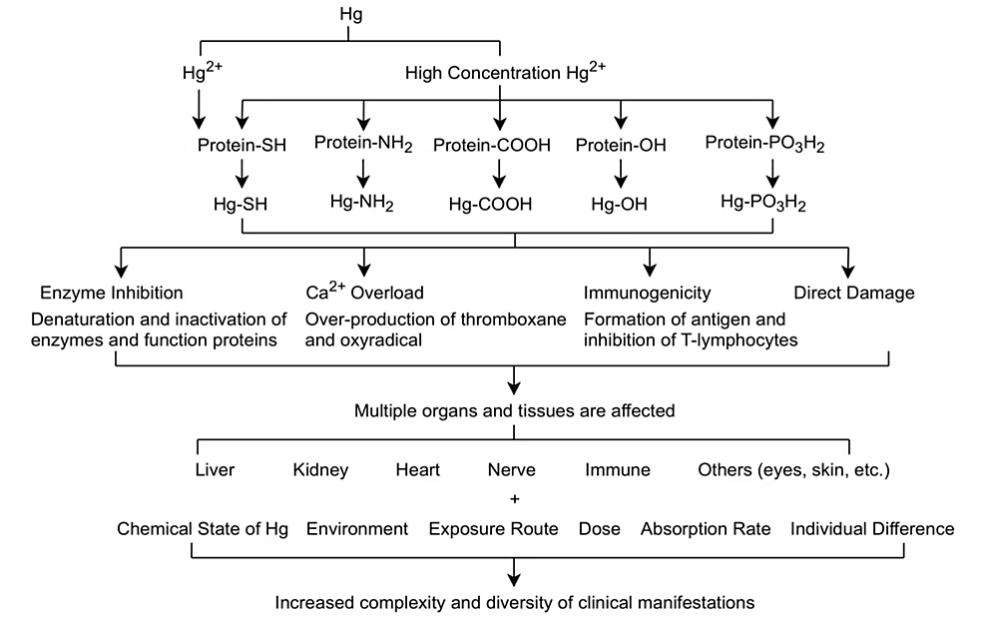
Proposed mechanisms of action of mercury toxicities.

There are multiple mechanisms of toxic action proposed for mercury, including upregulating of vascular endothelial growth factor expression within astrocytes, accumulating of intracellular calcium, affecting cell division, causing immune system disorders and neurodegenerative diseases [2,4-7]. The latency of sign and symptom manifestation ranges from months to years. Also, the mercury levels in the body may not correlate with the symptoms as the abnormal levels may develop no symptoms [8]. After reading literatures, we summarized the mercury poisoning mechanism as shown in Figure 3.

There have been more studies on central nervous system damage caused by mercury poisoning, and fewer studies on peripheral nervous system diseases [9]. Central nervous system damage is mainly manifested as mental and behavioral disorders, such as mental decline, ataxia, language disorders, hearing and vision disorders. In contrast, peripheral nervous system involvement is more common in elemental mercury and inorganic mercury poisoning, which is manifested as sensory disturbances in the limbs, with long-term, severe, spontaneous tingling or burning pain in the muscles [10,11]. It has been reported that pain is the main manifestation of mercury poisoning in patients. Research has shown that Glial cells’ activation is the key factor in the occurrence, development, and persistence of neuropathic pain. When peripheral nerves are injured, astrocytes are activated, and the expression of a glial fibrillary acidic protein (GFAP) is up-regulated. The neuroactive substances and pro-inflammatory cytokines released by glial cells act on the painful nerve cells in the dorsal horn of the spinal cord to induce pain and enhance the sensitivity and responsiveness of post-synaptic pain transmitting nerve cells to form hyperalgesia and persistent pain. Sodium dimercaptopropane sulfonate (DMPS) combined with pentoxifylline (POF) can significantly inhibit the activation of astrocytes [12-14]. Electrophysiological examination shows that the nerve conduction velocity slows down, and the incubation period prolongs both sensory and motor nerve. However, some patients wouldn’t show abnormalities in the peripheral nerve electrophysiological examination. Therefore, it has been speculated that the paranesthesia caused by mercury poisoning wouldn’t induce peripheral nerve damage, but central nervous system damage [15,16]. This patient had been poisoned for a long time and suffered from severe limb pain and fatigue, but the muscle enzymes were normal, and the electromyography and nerve conduction had no characteristic findings. It is worthy of attention. In recent years, most of the cases of chronic mercury poisoning with pain as the main symptom were caused by exposure to whitening cosmetics or folk remedies. When patients have unexplained pain, such as low back pain, limb pain, joint pain, they should be suspicious of the possibility of chronic mercury poisoning [17].

Laboratory tests for mercury poisoning should include blood cell analysis, serum electrolytes, liver and kidney function tests, urinalysis, and urine and blood mercury level. Patients with central nervous system toxicity should undergo neurological tests and clinical psychological evaluation. Since the mercury concentration in the blood tends to return to normal within a few days after exposure, blood samples are mainly used for short-term and high-level exposure. The concentration of mercury in the tissue is much higher than that in the blood, and the elimination half-life is about 70 days. In the case of a low concentration of blood mercury, the damage caused by the higher concentration of mercury in the tissue continues [18].

After mercury poisoning is diagnosed, mercury removal therapy should be performed in time. The first choice is the metal chelating agent, sodium dimercaptopropane sulfonate. Studies have found that urine microalbumin increases after using chelating agents suggesting that more mercury excreted through the kidneys may aggravate kidney damage, so chelating agents should be used intermittently, and attention should be paid to monitoring and protecting renal function during the treatment of mercury. Blood purification is mostly used for patients with acute mercury poisoning. The massive activation of astrocytes is related to the pain caused by mercury poisoning, and pentoxifylline can effectively inhibit the activation of astrocytes. Therefore, some studies suggest that sodium dimercaptopropane sulfonate combined with pentoxifylline could effectively reduce and alleviate pain. Its effectiveness still needs further verification.

## Conclusions

We report a case of mercury intoxication from daily cosmetics use. When patients present with unexplained nonspecific symptoms such as behavioral changes, cognitive decline, sleep disturbance, fatigue, pain, etc., the possibility of mercury poisoning should be considered, blood mercury and urine mercury should be checked, and the source of exposure should be investigated, and timely treatment should be given.
